# Supplementary material for: Dkk2 promotes neural crest specification by activating Wnt/β-catenin signaling in a GSK3β independent manner
Source: eLife. 2018 Jul 23;7:e34404. doi: 10.7554/eLife.34404 (PMC6056231; doi:10.7554/eLife.34404)
Supplement: Figure 1—source data 1. [file elife-34404-fig1-data1.docx]

| **Injection** | **Concentration** | **Probe** | **Phenotype** | | | **Total** |
| --- | --- | --- | --- | --- | --- | --- |
|  |  |  | **Normal** | **Reduced** | **Expanded** |  |
| Dkk2MO | 30ng | *snai2* | 4 | 38 | 2 | 44 |
|  |  | *sox10* | 2 | 46 | - | 48 |
|  |  | *sox2* | 3 | - | 31 | 34 |
| Dkk2SMO | 30ng | *snai2* | - | 244 | - | 244 |
|  |  | *sox10* | - | 38 | - | 38 |
|  |  | *sox2* | - | - | 30 | 30 |
|  |  | *dct* | - | 45 | - | 45 |

| **Injection** | **Concentration** | **Craniofacial structures** | | | **Total** |
| --- | --- | --- | --- | --- | --- |
|  |  | **Normal** | **Reduced** | **Expanded** |  |
| Dkk2SMO | 20ng | 8 | 32 | - | 40 |
