# Supplementary material for: Dkk2 promotes neural crest specification by activating Wnt/β-catenin signaling in a GSK3β independent manner
Source: eLife. 2018 Jul 23;7:e34404. doi: 10.7554/eLife.34404 (PMC6056231; doi:10.7554/eLife.34404)
Supplement: Figure 2—source data 1. [file elife-34404-fig2-data1.docx]

| **Injection** | **Concentration** | **Probe** | **Phenotype** | | | | **Total** |
| --- | --- | --- | --- | --- | --- | --- | --- |
|  |  |  | **Normal** | **Reduced** | **Shifted** | **Expanded** |  |
| Dkk2SMO | 20ng | *pax3* | - | 5 | 51 | - | 56 |
|  |  | *sox8* | 15 | - | 9 | - | 24 |
|  |  | *snai1* | 24 | - | 19 |  | 43 |
|  |  | *sox9* | 4 | 7 | 12 | - | 23 |
|  |  | *twist1* | - | 39 | - | - | 39 |
|  |  | *krt* | 9 | 12 | - | - | 21 |
|  |  | *myod* | 43 | - | - | - | 43 |
|  |  | *actc1* | 30 | - | - | - | 30 |
|  |  | *pcdh8* | 14 | - | 17 | - | 31 |
