# Supplementary material for: Dkk2 promotes neural crest specification by activating Wnt/β-catenin signaling in a GSK3β independent manner
Source: eLife. 2018 Jul 23;7:e34404. doi: 10.7554/eLife.34404 (PMC6056231; doi:10.7554/eLife.34404)
Supplement: Figure 5—source data 1. [file elife-34404-fig5-data1.docx]

| **Injection** | **Concentration** | **Probe** | **Phenotype** | | | **Total** |
| --- | --- | --- | --- | --- | --- | --- |
|  |  |  | **Normal** | **Reduced** | **Expanded** |  |
| Dkk2SMO | 30ng | snai2 | 2 | 23 | 2 | 27 |
| β-catenin DNA | 50pg |  | 9 | - | 30 | 39 |
| Dkk2SMO+  β-catenin | 30ng+50pg |  | 23 | 15 | 18 | 56 |
| Dkk2SMO | 30ng |  | 7 | 18 | - | 25 |
| Lrp6 DNA | 50pg |  | 2 | - | 52 | 54 |
| Dkk2SMO+  Lrp6 DNA | 30ng+50pg |  | 22 | 8 | 22 | 52 |
| Dkk2SMO | 30ng |  | - | 32 | - | 32 |
| Wnt8 DNA | 200pg |  | - | - | 58 | 58 |
| Dkk2SMO+  Wnt8 DNA | 30ng+200pg |  | - | 49 | 14 | 63 |
