# Supplementary material for: Dkk2 promotes neural crest specification by activating Wnt/β-catenin signaling in a GSK3β independent manner
Source: eLife. 2018 Jul 23;7:e34404. doi: 10.7554/eLife.34404 (PMC6056231; doi:10.7554/eLife.34404)
Supplement: Figure 6—source data 1. [file elife-34404-fig6-data1.docx]

| **Injection** | **Concentration** | **Probe** | **Phenotype** | | | **Total** |
| --- | --- | --- | --- | --- | --- | --- |
|  |  |  | **Normal** | **Reduced** | **Expanded** |  |
| dkk2 mRNA | 500pg | *snai2* | 7 | 2 | 34 | 43 |
| dkk2 DNA | 25pg |  | - | - | 122 | 122 |
| dkk2 DNA  (zebrafish) | 50pg |  | 21 | 2 | 54 | 77 |
| DKK2 DNA  (human) | 50pg |  | 23 | - | 41 | 64 |
| Dkk1 DNA | 50pg |  | 5 | 37 | 1 | 43 |

| **Injection** | **Concentration** | **Probe** | **Phenotype** | | | **Total** |
| --- | --- | --- | --- | --- | --- | --- |
|  |  |  | **Normal** | **Reduced** | **Expanded** |  |
|  |  | *myod*  *actc1*  *pcdh8* | 31 | - | - | 31 |
| dkk2 DNA | 50pg |  | 67 | - | - | 67 |
|  |  |  | 50 | - | - | 50 |
