# Supplementary material for: Dkk2 promotes neural crest specification by activating Wnt/β-catenin signaling in a GSK3β independent manner
Source: eLife. 2018 Jul 23;7:e34404. doi: 10.7554/eLife.34404 (PMC6056231; doi:10.7554/eLife.34404)
Supplement: Figure 7—source data 1. [file elife-34404-fig7-data1.docx]

| **Injection** | **Concentration** | **Probe** | **Phenotype** | | | **Total** |
| --- | --- | --- | --- | --- | --- | --- |
|  |  |  | **Normal** | **Reduced** | **Expanded** |  |
| Dkk2 DNA | 50pg | *snai2* | - | - | 40 | 40 |
| β-cateninMO | 20ng |  | - | 57 | - | 57 |
| Dkk2+  β-cateninMO | 50pg+20ng |  | - | 32 | - | 32 |
| Lrp6MO | 20ng |  | - | 51 | - | 51 |
| Dkk2+  Lrp6MO | 50pg+20ng |  | - | 68 | - | 68 |
| Wnt8MO | 40ng |  | 7 | 46 | - | 53 |
| Dkk2+  Wnt8MO | 50pg+40ng |  | 6 | 44 | - | 50 |
