# Supplementary material for: Dkk2 promotes neural crest specification by activating Wnt/β-catenin signaling in a GSK3β independent manner
Source: eLife. 2018 Jul 23;7:e34404. doi: 10.7554/eLife.34404 (PMC6056231; doi:10.7554/eLife.34404)
Supplement: Figure 8—source data 1. [file elife-34404-fig8-data1.docx]

| **Injection** | **Concentration** | **Probe** | **Phenotype** | | | **Total** |
| --- | --- | --- | --- | --- | --- | --- |
|  |  |  | **Normal** | **Reduced** | **Expanded** |  |
| BIO | 10μM | *sox10* | 46 | - | 142 | 188 |
| Dkk2SMO | 30ng |  | - | 45 | - | 45 |
| Dkk2SMO+  BIO | ng+10μM |  | 3 | 60 | 8 | 71 |
| Dkk1 | 50pg |  | - | 45 | - | 45 |
| Dkk1+  BIO | 50pg+10μM |  | - | 10 | 45 | 55 |
