# Supplementary material for: Dkk2 promotes neural crest specification by activating Wnt/β-catenin signaling in a GSK3β independent manner
Source: eLife. 2018 Jul 23;7:e34404. doi: 10.7554/eLife.34404 (PMC6056231; doi:10.7554/eLife.34404)
Supplement: Transparent reporting form [file elife-34404-transrepform.docx]

***eLife’s* transparent reporting form**

We encourage authors to provide detailed information *within their submission* to facilitate the interpretation and replication of experiments. If you have any questions, please contact us: [editorial@elifesciences.org](mailto:editorial@elifesciences.org).

**Sample-size estimation**

- You should state whether an appropriate sample size was computed when the study was being designed
- You should state the statistical method of sample size computation and any required assumptions
- If no explicit power analysis was used, you should describe how you decided what sample (replicate) size (number) to use

Appropriate numbers of embryos to be analyzed were determined based on robustness of phenotypes across multiple biological replicates (different clutch of embryos obtained from different mothers). This information is included in the material and methods section (page 15).

Please outline where this information can be found within the submission (e.g., page numbers or figure legends), or explain why this information doesn’t apply to your submission:

**Replicates**

- You should report how often each experiment was performed
- You should include a definition of biological versus technical replication
- The data obtained should be provided and sufficient information should be provided to indicate the number of independent biological and/or technical replicates
- If you encountered any outliers, you should describe how these were handled
- Criteria for exclusion/inclusion of data should be clearly stated
- High-throughput sequence data should be uploaded before submission, with a private link for reviewers provided (these are available from both GEO and ArrayExpress)

For whole embryo experiments more than 30 embryos and for animal cap experiments 10-12 animal caps per conditions were used and experiments were repeated on at least 3 different biological replicates (different clutch of embryos obtained from different mothers). This information is included in the material and methods section (page 15).

Information about the total number of embryos analyzed for each injection/treatment can be found on the top of each bar in the graphs providing quantification of the phenotypes (Fig 1, 3, 4, 5 and 6)

For in-situ hybridization experiments only embryos with co-localized expression of the lineage tracer with the cell type marker were considered for analysis. This information is included in the material and methods section (page 14-15).

For qRT-PCR, the mean Ct value for each biological replicate was obtained from 4 experimental replicates. To compute the p value for the Student’s t-test we used the mean from 3 different biological replicates. This information is included in the material and methods section (page 15).

Please outline where this information can be found within the submission (e.g., page numbers or figure legends), or explain why this information doesn’t apply to your submission:

**Statistical reporting**

- Statistical analysis methods should be described and justified
- Raw data should be presented in figures whenever informative to do so (typically when N per group is less than 10)
- For each experiment, you should identify the statistical tests used, exact values of N, definitions of center, methods of multiple test correction, and dispersion and precision measures (e.g., mean, median, SD, SEM, confidence intervals; and, for the major substantive results, a measure of effect size (e.g., Pearson's r, Cohen's d)
- Report exact p-values wherever possible alongside the summary statistics and 95% confidence intervals. These should be reported for all key questions and not only when the p-value is less than 0.05.

The statistical analysis method used for qRT-PCR was computed using pairwise, two-tailed Student’s t-test with p value less than 0.05 considered significant. The computed p values are described in the figure legends (Fig 2). The Student’s t-test was computed using the mean from at-least 3 biological replicates (different clutch of embryos obtained from different mothers).

Please outline where this information can be found within the submission (e.g., page numbers or figure legends), or explain why this information doesn’t apply to your submission:

(For large datasets, or papers with a very large number of statistical tests, you may upload a single table file with tests, Ns, etc., with reference to page numbers in the manuscript.)

**Additional data files (“source data”)**

- We encourage you to upload relevant additional data files, such as numerical data that are represented as a graph in a figure, or as a summary table
- Where provided, these should be in the most useful format, and they can be uploaded as “Source data” files linked to a main figure or table
- Include model definition files including the full list of parameters used
- Include code used for data analysis (e.g., R, MatLab)
- Avoid stating that data files are “available upon request”

Please indicate the figures or tables for which source data files have been provided:
